# One Health defines an emerging sixth wave of public health development

**DOI:** 10.7189/jogh.13.03062

**Published:** 2023-11-29

**Authors:** Jesus D Cortes Gil, Pedro M Vargues Aguiar, Paulo Ferrinho

**Affiliations:** 1 NOVA National School of Public Health, Public Health Research Centre, Comprehensive Health Research Centre, CHRC, NOVA University Lisbon, Lisbon, Portugal; 2 Global Health and Tropical Medicine, Instituto de Higiene e Medicina Tropical, Universidade Nova de Lisboa, Lisbon, Portugal

**Figure:**
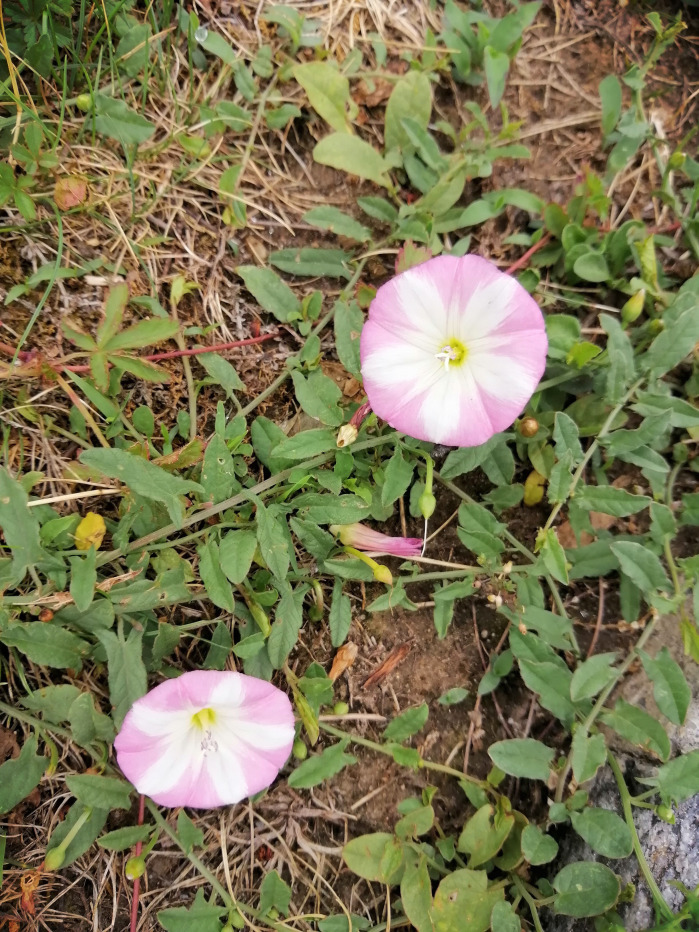
Photo: Suumer Flower Lisbon, Portugal, 2023. Source: Authors’ personal collection.

According to researchers, there have been five waves in public health development over the years, conceptualised “according to the focus of approach taken” as structural (1830-1900), biomedical (1890-1950), clinical (1940–1980), social (1960-2015), and cultural (2010-present). These labels are indicative of activity characterising each wave [1].

Here we argue that the growing acknowledgment of One Health as “an integrated, unifying approach that aims to sustainably balance and optimize the health of people, animals, and ecosystems” and that recognizes “the health of humans, domestic and wild animals, plants, and the wider environment (including ecosystems)” as “closely linked and interdependent”, demanding an approach that “mobilizes multiple sectors, disciplines, and communities at varying levels of society to work together to foster well-being and tackle threats to health and ecosystems while addressing the collective need for healthy food, water, energy, and air, taking action on climate change and contributing to sustainable development” [2], calls for the definition of a 6^th^ wave of public health.

The One Health High Level-Expert Panel (OHHLEP) posits that a sustainable future revolves around essential principles such as equity, parity, socio-ecological balance, transdisciplinary, and stewardship; this last one encompasses the responsibility of human beings to change and adopt sustainable solutions to emerging problems [3].

Over the past two centuries, the previous five waves helped greatly improve planetary health. As scientific knowledge grew, public agencies expanded their interventions to include sanitation, immunisation, regulation, health education, promotion, and protection, and universal access to personal health care. Major scourges such as smallpox, polio, and measles were eliminated or contained.

Nonetheless, planetary health continues to face serious public health challenges related to the anthropocentric-driven search for pleasure, comfort, sophistication, and power, interfering with and altering the climate and the environment and threatening the living conditions and survival of Homo Sapiens and many other species.

Several recent zoonotic disease outbreaks exemplify this, the most recent being the coronavirus disease 2019 (COVID-19) pandemic, which has mobilised an unprecedented global response. These overlap with the growing threat of human and non-human non-communicable diseases [4] and silent public health emergencies such as antimicrobial resistance [5].

The pandemic has been considered an outcome of an often neglected and degraded complex planetary system [6], which comprises climate change, pollution, land use, food markets, poverty, population growth, and low levels of effective governance, among other factors [6]. The ongoing or future massive international and internal migrations and potential conflicts in today’s complex, dynamic world also play an important role in public health.

This syndemic complex highlights the relevance of One Health. The narratives of the previous five waves did not sufficiently emphasize the symbiotic relationships between human, animal, plant, and environmental health, infectious and non-infectious diseases. We seek to highlight that, although the idea of One Health can be useful for limiting the spread of new emerging and re-emerging infections, epidemics, pandemics, and non-communicable diseases, it requires significant changes in current cultural, social, economic, and health (human, animal, plant and environmental) systems and practices to be implemented successfully.

COVID-19 has forced an often creative, large-scale response from social and health care systems in countries worldwide and highlighted challenges that will have to be resolved in the future. The pandemic laid the basis for defining a sixth wave of public health development, conditional on accepting the centrality of the One Health approach.

After this crisis is resolved, translating One Health into action will be an urgent global goal [7].
